# The magnitude and determinants of depressive symptoms amongst women in early pregnancy in Southern Nigeria: A cross-sectional study

**DOI:** 10.4102/sajpsychiatry.v28i0.1691

**Published:** 2022-05-31

**Authors:** Omolola F. Oboro, Vincent Ebulue, Victor O. Oboro, Victor Ohenhen, Adeoye Oyewole, Rasaq Akindele, Olufemi Ala, Olaolu Oyeniran, Adegboye Isawumi, Babatunde Afolabi

**Affiliations:** 1 Perinatal Mental Health Unit, OMVIAL Medical Group, Benin-City, Nigeria; 2 Department of Obstetrics and Gynaecology, Central Hospital Benin, Benin-City, Nigeria; 3 Department of Psychiatry, Faculty of Clinical Sciences, Ladoke Akintola University, Ogbomoso, Nigeria; 4 Department of Obstetrics and Gynaecology, LAUTECH Teaching Hospital, Osogbo, Nigeria; 5 Department of General Practice, LAUTECH Teaching Hospital, Osogbo, Nigeria

**Keywords:** depression, pregnancy, antenatal, magnitude, determinants

## Abstract

**Background:**

Antenatal depression (AD) is prevalent and associated with adverse pregnancy, maternal and child outcomes, yet no study has addressed its magnitude and predictors in early pregnancy in Nigeria.

**Aim:**

To determine the prevalence and factors associated with AD in first half of pregnancy.

**Setting:**

Multicentric health facilities in Southern Nigeria.

**Methods:**

A multicentric health-facilities-based cross-sectional study was conducted from January to July 2018. Using pretested structure interviewer-administered questionnaires, antenatal depressive symptoms were assessed amongst 511 pregnant mothers with the Edinburg Postnatal Depressive Scale tool. Socio-demographic, socio-economic, clinical, family and social factors were also measured. Descriptive statistics, bivariate and multivariable logistic regression analyses were employed to describe and identify factors associated with AD.

**Results:**

The prevalence of antenatal depressive symptoms in early pregnancy in this study was 29.4% (95% confidence interval [CI] 26.6–32.9). Factors independently associated with AD were intimate partner violence (adjusted odds ratios [AOR] = 8.10, 95% CI 5.00–13.14), marital dissatisfaction (AOR 5.48, 95% CI 3.48–8.38), poor social support (AOR 4.70; 95% CI 2.99–7.38), past history of depression (AOR 4.67; 95% CI 2.47–8.80), previous pregnancy complication (AOR 2.50, 95% CI 1.57–3.89), low socio-economic status (AOR 2.41, 95% CI 1.61–3.66) and unplanned pregnancy (AOR 2.35, 95% CI 1.47–3.64).

**Conclusions:**

The prevalence of antenatal depression is high with modifiable risk factors requiring context-specific policies such as provision of family, social and economic support for mothers at the earliest possible contact in the antenatal period.

## Introduction

Antenatal depression (AD) is a major depressive episode occurring in pregnancy. Pregnancy is a major life event accompanied by psychological and physiological changes,^1^ which increase pregnant women’s vulnerability for the onset or recurrence of mental disorders.^2^ Antenatal depression has been described as one of the least investigated and under-addressed medical conditions.^3^

The prevalence of depression is high with global estimates of about 17% in the perinatal period, and ranging from 0.5% to 51% in the antenatal period.^3^ The prevalence appears to be trimester-specific, with reported figures from developed countries being 7%, 13% and 12% in the first, second and third trimesters of pregnancy, respectively.^4^ In low- and middle-income countries (LMICs), the prevalence is even higher, ranging from between 8% and 78% in different African countries.^5^ It is reported that perinatal depression is underdiagnosed^6^ and there are significant heterogeneities even across countries with similar socio-economic circumstances.^7^

The burden of AD is varied, and include: (1) adverse impacts on mother (women with AD are at higher risk for substance abuse, preeclampsia, oedema, premature rupture of membranes, haemorrhage and other adverse pregnancy complications), (2) impact on infant’s health including preterm birth or low birth weight, but also adverse impact on their neurological, behavioural and emotional development, (3) postnatal depression (women who suffered from AD are more likely to develop postnatal depression^8^ bipolar depression^6,7,8^) and (4) high health and economic cost, including life years lived with disability of up to 6%.^9,10^

The aetiology of depression in pregnancy is unclear, but multiple risk factors have been highlighted in systematic reviews, and these include poor social support, unplanned pregnancy, history of depression, unemployed status and financial problems.^10^ However, there have been no agreement among authors regarding what constitute risk factors, hence the lack of decision makers’ policy attention to perinatal mental health problems, especially in LMICs. In Nigeria, only two studies have so far been carried out on AD,^11,12^ both of which predominantly contain data for the third trimester of pregnancy. There is therefore the need for a study to (1) address the lack of local data, (2) identify factors, unique to Nigeria, which are associated with depression, (3) design intervention for early identification and treatment and (4) reduce the burden of AD.

The aim of the present study was to determine, amongst women presenting for antenatal services in Southern Nigeria, the prevalence of AD and, if any, socio-demographic, obstetric and social support characteristics were associated with depression.

## Materials and methods

### Study design and period

This was a prospective, cross-sectional study, conducted between January 2018 and July 2018.

### Study setting

The southern part of Nigeria comprises half of the six geopolitical zones of the country, namely the North Central (NC), North East (NE), North West (NW), South West (SW), South East (SE) and South (SS). The study settings were selected to provide a fair representation of the national population.

### Study participants

The Source population for this study was antenatal women in Southern Nigeria. The study population were pregnant women presenting for antenatal services in any of the health facilities of Omvial Medical Group. Participants were included in the study if they were in the first half of pregnancy (<20 weeks), aged 18 years or above, and able to understand the content of the questionnaire. Exclusion criteria were critically ill women and women currently undergoing mental health interventions.

### Sample size

Sample size was determined using an online single population proportion calculator (https://www.benchmarksixsigma.com/calculators/sample-size-estimationproportion-data/) with an assumption of 95% confidence interval (CI), a prevalence of 24.5% of AD in Nigeria,^9^ a 5% margin of error and a nonresponse rate of 10%, to arrive at a size of 511.

### Data collection

Data were collected by means of a pretested, face-to-face interviewer-administered structured questionnaire. The questionnaire was prepared first in English and translated into Yoruba and Pidgin English and then back to English by two different language experts and psychiatrists to check for consistency. The three versions of the questionnaire were used to collect the data according to participants’ preference. The questionnaire was pretested on randomly selected 5% of the sample size and amended accordingly before implementation for this study. The questionnaire included four sections: socio-demographic characteristics, obstetric, family and social factors and Edinburgh Postnatal Depression Scale (EPDS). The socio-demographic characteristics included age, educational status, ethnicity, residence, current marital status, religion, socio-economic status, occupational status and average monthly income. Obstetric factors included gravidity, history of miscarriage, preterm delivery or stillbirth, previous pregnancy complications, antenatal visit (defined as having booked for antenatal care the current pregnancy), current pregnancy planning, current pregnancy complications (e.g. threatened miscarriage, medical disorders, etc.), previous mode of delivery (operative or caesarean), previous history of depression and substance use (e.g. alcohol, tobacco, illicit drugs) and foetal sex preference. Family and social support factors included intimate partner violence (IPV) in all its form, marital satisfaction and feeling of partner or family or friend support. Marital satisfaction was assessed as a ‘yes’ or ‘no’ response to the question: ‘are you satisfied with your marriage?’ Partner or family or friend support was assessed with three questions, namely, ease of obtaining needed help, number of close people who can be counted upon in time of trouble and how much concern others have shown in their interest. These answers were scored, and scores less than nine were taken as poor social support.^13^

The EPDS was developed by Cox et al. in 1987 as a screening tool for postnatal depression and does not give a definitive diagnosis of depression.^14^ It is a simple, free to use tool with a sensitivity and specificity of 84.6% and 77.0% respectively, and a Cronbach’s reliability coefficient of 0.85 (a Cronbach’s alpha of 0.70 is the cut-off for reliability in most behavioural research studies). The EPDS contains 10 items, each of which describes a specific symptom of depression with a four-point Likert-scale response option (‘most of the time’, ‘sometimes’, ‘not often’, ‘never’) scored 0–3, with a total score ranging from 0 to 30. The cut-off point of 10 was used to identify pregnant women with depressive symptoms.^19^ The EPDS is the most commonly used screening tool in pregnancy because it does not include the somatic symptoms of pregnancy such as nausea, headache, appetite or weight changes that are all symptoms associated with depression and may result in higher depression scores.^15,16^ It is also particularly recommended for low-resource settings for screening for perinatal depression because of its potential to detect common perinatal mental disorders in culturally diverse low-income and lower middle-income countries because its validity and reliability has been studied in these settings and found to be acceptable.^17^

Research assistants, who were trained on the study objective, the data collection process andadministration of the EPDS, collected the data.

### Data analysis

Data were coded and exported into Statistical Package for the Social Sciences (SPSS) for recoding, categorisation and statistical analysis. Data were summarised using descriptive statistics such as frequency and percentages for categorical data, and mean +/- standard deviation (SD) and range for numerical data. Bivariate analysis was carried out to estimate the association between potential risk factors (independent variables) and AD (dependent variable). Risk factors with significant association with AD at *p* < 0.05 were included in a multivariate logistic regression analysis to identify independent risk factors for AD. Estimated associations were described using crude and then adjusted odds ratios (AOR) with 95% CIs. The SPSS 25.0 (SPSS, Chicago, IL, USA) was used for all statistical analyses.

### Ethical considerations

The Ethical Committee on Human Research of OMVIAL Group of Hospitals, OMVIAL Nigeria Ltd., (a private organisation), approved the study. Written informed consent was first obtained from all the participants.

## Results

A total of 511 out of 550 invited pregnant women completed the questionnaire, resulting in a response rate of 93%.

On screening with the EPDS tool, 150 participants met the cut-off point of 10 that was used to identify pregnant women with depressive symptoms, resulting in a prevalence rate of 29.4%. The socio-demographic, obstetric, family and social support characteristics of the study population, the depressed and non-depressed groups are shown in Tables 1 and 2, respectively.

**TABLE 1:** Socio-demographic characteristics of pregnant women in early pregnancy.

Variables	Categories	Study population		Depressed group		Non-depressed group		Significance	
		*n*	%	*n*	%	*n*	%	χ^2^	*p*
Maternal age (in years)	< 25	213	41.7	73	34.3	140	65.7	4.281	0.12
	25–34	234	45.8	60	25.6	174	74.4		
	35+	64	12.5	17	26.6	47	73.4		
Educational status	No formal	211	41.3	71	33.6	140	66.4	3.197	0.07
	Formal	300	58.7	79	26.3	221	73.7		
Ethnicity	Yoruba	154	30.1	49	31.8	105	68.2	0.647	0.72
	Igbo	315	61.6	89	28.3	226	71.7		
	Others	42	8.2	12	28.6	30	71.4		
Residence	Rural	171	33.5	59	34.5	112	65.5	3.285	0.07
	Urban	340	66.5	91	26.8	249	73.2		
Current marital status	Unmarried*	141	27.6	44	31.2	97	68.8	0.322	0.57
	Married	370	72.4	106	28.6	264	71.4		
Religion	Christian	358	70.1	113	31.6	245	68.4	2.816	0.09
	Muslim	153	29.9	37	24.2	116	75.8		
Socioeconomic status	Low	154	30.1	73	47.4	81	52.6	34.771	0.00
	Medium	315	61.6	69	21.9	246	78.1		
	High	42	8.2	8	19.0	34	81.0		
Occupational status	Unemployed	149	29.2	49	32.9	100	67.1	1.265	0.26
	Employed	362	70.8	101	27.9	261	72.1		
Average monthly income (Naira)	< 50 000	223	43.6	74	33.2	149	66.8	2.798	0.09
	≥ 50 000	288	56.4	76	26.4	212	73.6		

* , Single, divorced, widowed.

**TABLE 2:** Obstetric, medical, social and family characteristics of the study population.

Variables	Categories	Study population		Depressed group		Non-depressed group		Significance	
		*n*	%	*n*	%	*n*	%	χ^2^	*p*
Gravidity	Primigravida	161	31.5	51	31.7	110	68.3	0.612	0.430
	Multigravida	350	68.5	99	28.3	251	71.7		
History of miscarriage	Yes	94	18.4	33	35.1	61	64.9	1.838	0.180
	No	417	81.6	117	28.1	300	71.9		
History of preterm delivery	Yes	68	13.3	24	35.3	44	64.7	1.335	0.250
	No	443	86.7	126	28.4	317	71.6		
History of stillbirth	Yes	60	11.7	24	40.0	36	60.0	3.715	0.050
	No	451	88.3	126	27.9	325	72.1		
Previous pregnancy complications	Yes	102	20.0	50	49.0	52	51.0	23.765	< 0.001
	No	409	80.0	100	24.4	309	75.6		
Antenatal visits	Yes	375	73.4	104	27.7	271	72.3	1.785	0.180
	No	136	26.6	46	33.8	90	66.2		
Current pregnancy unplanned	Yes	122	23.9	55	45.1	67	54.9	19.116	< 0.001
	No	389	76.1	95	24.4	294	75.6		
Current pregnancy complication	Yes	143	28.0	44	30.8	99	69.2	0.192	0.660
	No	368	72.0	106	28.8	262	71.2		
Operative vaginal delivery	Yes	101	19.8	36	35.6	65	64.4	2.401	0.120
	No	410	80.2	114	27.8	296	72.2		
Caesarean delivery	Yes	138	27.0	49	35.5	89	64.5	3.452	0.060
	No	373	73.0	101	27.1	272	72.9		
Foetal gender preference	Yes	158	30.9	70	44.3	88	55.7	24.649	< 0.001
	No	353	69.1	80	22.7	273	77.3		
Substance use	Yes	59	11.5	23	39.0	36	61.0	2.982	0.080
	No	452	88.5	127	28.1	325	71.9		
IPV	Yes	110	21.5	73	66.4	37	33.6	92.584	< 0.001
	No	401	78.5	77	19.2	324	80.8		
Past history of depression	Yes	51	10.0	32	62.7	19	37.3	30.460	< 0.001
	No	460	90.0	118	25.7	342	74.3		
Marital dissatisfaction	Yes	109	21.3	65	59.6	44	40.4	61.255	< 0.001
	No	402	78.7	85	21.1	317	78.9		
Social support	Poor	116	22.7	66	56.9	50	43.1	54.894	< 0.001
	Good	395	77.3	84	21.3	311	78.7		

IPV, intimate partner violence.

The mean (±SD) age of the study population was 27.9 (± 6.01) years with a range of 18–43 years, with most of the respondents (234, 46%) being aged between 25 and 34 years, and more than half (300, 59%) had formal education. The majority of the respondents were Igbos (315, 62%), lived in urban areas (340, 67%), were married (370, 72%) and practiced Christianity (358, 70%). Two-third of the respondents (62%) were of medium socio-economic status, majority (362, 71%) were employed and more than half (56.4%) earned more than 50 000 naira monthly (Table 1). Regarding obstetric characteristics, most of the respondents were multigravida (350, 69%), had no history of miscarriage (417, 82%), had a preterm delivery (443, 87%), still birth (451, 88%) or previous pregnancy complication (80%). Most of the participants were booked for antenatal visits and had the index pregnancy planned 375 (73%) and 389 (76%), respectively. Approximately one-quarter (143, 28%) of the total respondents had current pregnancy complications, with a fifth having a history of operative vaginal delivery, and about a quarter having a history of previous caesarean delivery, 101 (20%) and 138 (27%), respectively. Almost one-third (158, 31%) of the respondents, showed foetal gender preference. Substance use was reported by 59 (12%) of the respondents, and IPV was reported in more than one-fifth (110, 22%) of the women (Table 2).

Bivariable analysis showed that there was a statistically significant association (*p* < 0.05) between poor socio-economic status, previous pregnancy complications, unplanned current pregnancy, past history of depression, IPV, marital dissatisfaction, substance use, poor social support and foetal gender preference.

The results of multivariate analysis are shown in Table 3. Participants risk exposure of developing AD was found to be as follows:

- who were exposed to IPV were at eight times greater risk (AOR = 8.10, 95% CI 5.00–13.14);
- who reported marital dissatisfaction at five times greater risk (AOR 5.48, 95% CI 3.48–8.38);
- who had low social support were at five times greater risk (AOR 4.70; 95% CI 2.99–7.38);
- who had past history of depression at five times greater risk (AOR 4.67; 95% CI 2.47–8.80);
- who reported previous pregnancy complication were at three times greater risk (AOR 2.77, 95% CI 1.78–4.47);
- who showed foetal gender preference were at three times greater risk (AOR 2.71, 95% CI 1.82–4.05);
- who had low socio-economic status were at two times greater risk (AOR 2.41, 95% CI 1.61–3.66); and
- whose pregnancy was unplanned were at two times greater risk (AOR 2.35, 95% CI 1.47–3.64, respectively).

**TABLE 3:** Multivariate analysis.

Variables	Categories	Depressed		Non-depressed		cOR	95%	CI
		*n*	%	*n*	%			
Socio-economic status	Low	73	47.4	81	52.6	3.21	2.12	4.86
	Medium	69	21.9	246	78.1	1.00		
	High	8	19.0	34	81.0	0.84	0.37	1.36
Previous pregnancy complications	Yes	50	49.0	52	51.0	2.97	1.90	4.65
	No	100	24.4	309	75.6	1.00		
Current pregnancy unplanned	Yes	55	45.1	67	54.9	2.54	1.66	3.89
	No	95	24.4	294	75.6	1.00		
Foetal gender preference	Yes	70	44.3	88	55.7	2.71	1.82	4.05
	No	80	22.7	273	77.3	1.00		
IPV	Yes	73	66.4	37	33.6	8.10	5.20	13.24
	No	77	19.2	324	80.8	1.00		
Past history of depression	Yes	32	62.7	19	37.3	4.88	2.67	8.94
	No	118	25.7	342	74.3	1.00		
Marital dissatisfaction	Yes	65	59.6	44	40.4	5.51	3.51	8.65
	No	85	21.1	317	78.9	1.00		
Poor social support	Poor	66	56.9	50	43.1	4.89	3.15	7.58
	Good	84	21.3	311	78.7	1.00		

IPV, intimate partner violence; CI, confidence interval; cOR, corrected Odd Ratio.

## Discussion

The prevalence rate of antenatal depressive symptoms in early pregnancy in this study was 29.4% (95% CI 26.6–32.9). This was consistent with previous studies such as from North Ethiopia (31.1%),^18^ Adama, Ethiopia (31.2%),^19^ Bangladesh^20^ and Nairobi, Kenya (32.9%).^21^ but higher than the findings from South Ethiopia (23.3%),^22^ Vietnam (25%),^23^ South Ethiopia (16.3%)^24^ and North West Ethiopia (15.2).^25^ However, higher findings of up to 80% have been reported from Pakistan.^26,27^ The variations in prevalence figures could be because of differences in the socio-cultural settings, tools used in assessment of depression (along with cut-off scores, e.g., EPDS score of 13 was used as cut-off in some studies),^28,29^ trimester of pregnancy^30,31^ and differences across populations other than those mentioned above.^5^ Nevertheless, pooled prevalence from systematic review of systematic reviews put the prevalence figures from 6% to 56%,^32^ supporting the fact that the prevalence of depression amongst pregnant women is high.

A systematic review by Biaggi et al.^10^ reported multiple factors that are associated with AD such as poor partner or social support, history of abuse or domestic violence, history of mental illness, unintended pregnancy and present or past pregnancy complications. This study found that the odds of developing AD were about eight times higher amongst women who reported IPV. This is in line with previous studies from Ethiopia,^20^ Malawi^33^ and Bangladesh.^34^ This could be explained by the inherent humiliating nature of violence,^33^ especially during times of pregnancy when the escape from partner from whom the closest support is desired is often difficult to contemplate.^35^ Whether pregnancy is a trigger for IPV is unclear as available evidence is conflicting,^36^ but a World Health Organization’s (WHO) multi-country study on women’s health and domestic violence against women reported that around half of the participants in three sites stated that they experienced physical IPV for the first time in pregnancy. Nevertheless, the strong association between IPV in pregnancy and AD raised the need for violent reduction interventions during antenatal care, one of such intervention, ‘empowerment counselling’, which incorporates danger risk assessment and safety plan has shown a decrease in psychological and physical well-being in various settings.^37,38^ Moreover, antenatal women irrespective of ethnical backgrounds look forward to antenatal care as a window of opportunity for disclosure of IPV, which should not be missed.^39^

The current study also identified that marital dissatisfaction was significantly associated with experiencing of AD with mothers who reported marital dissatisfaction in their current relationship being five times more likely to experience depressive symptoms when compared with their counterparts. This study finding is congruent with the study finding from previous reports.^34,40^ This might be because of the fact that women considered their partner their first source of satisfaction in life, especially during times of emotional instability.^29^ Indeed, it is believed that the adverse pregnancy outcomes associated with marital dissatisfaction were mediated by psychological distress, particularly depression and anxiety,^41^ and improving couple communication skills that emphasise needs of the pregnant women improves marital satisfaction and consequently psychological health of pregnant women.^41^ Furthermore, marital dissatisfaction is integrally linked to IPV, poor social support, low socio-economic status and unplanned pregnancy, and therefore should be a focus of programmatic intervention.

Women in the category of low socio-economic status were 2.5 more like to develop depressive symptoms in early pregnancy compared to those not in this category. This finding is consistent with reports from other studies.^1,32^ This is likely because socio-economically un-empowered women would be psychologically unprepared to cope with the added responsibility of pregnancy and worry about the attending financial consequences, predisposing the development of depressive symptoms.^42^

Unplanned pregnancy was associated with depressive symptoms in early half of pregnancy. Women who had not planned their pregnancy were 2.5 times more likely to have early AD as compared with those who had the current pregnancy planned. This finding is in line with cross-sectional studies conducted in Ethiopia,^27,43^ Brazil^44^ and Malaysia,^45^ and this could be explained by the fact that an unplanned pregnancy is more likely to be unwanted, often occurring in pre- or extra-marital settings, hence psychologically burdensome with attendant predisposition to development of depression.^46,47^

Another strong predictor for AD was social support level. The likelihood of experiencing antenatal depressive symptoms amongst women who reported low social support was nearly five times higher as compared to those who did not feel socially unsupported. The current study finding was congruent with findings from Ethiopia,^20^ Malawi^48^ and Nigeria.^8^ Support from friends and family is a source of empowerment to deal with the added stress of pregnancy and home responsibilities, hence would serve as a protective factor against depression.^49^ Poor social support is directly related to marital disharmony and IPV, which could pose a challenge to the objective evaluation of the support that a woman receives in pregnancy.^50^ Besides, the low level of support perceived by depressed women may be more subjective than real, and might actually be a signal for help in an otherwise disharmonious relationship.^51^

Foetal gender preference was significantly associated with antenatal depressive symptoms in the current study. Male foetal preferences increase the odds of AD three times. This is consistent with other studies.^52,53^ Nigeria is a predominantly patriarchal society with women being under undue pressure to produce male offsprings.^42^ This psychological pressure can precipitate depressive symptoms, especially as the support (partner, social and family) enjoyed by a woman is influenced by whether a woman bears male or a female children, in that a woman with a male offspring is often more appreciated than their counterparts bearing only female children.^54,55^

The pregnant women who had past history of depression were around five times more likely toexperience AD as compared to those who had no history of previous depression. This is consistent with study findings from other settings.^44,56^ This might be because of possible genetic predisposition of those pregnant women to emotional disturbance, hence vulnerable to development of depressive symptoms because of the added effect of hormonal changes in pregnancy.^6^ It is also possible that the history of mental health in prior pregnancy increases the detection rate of any mental health disorder in subsequent pregnancy as a result of increase in level of surveillance occasioned by the presence of previous history in order to intervene early.^1,57^

The current study also found that pregnant women with a previous history of obstetric complications were three times more likely to have AD compared to those without a history of past obstetric complications. This finding was in agreement with the previous reports,^22,43^ which indicated that previous and current obstetric complications significantly increase the likelihood of development of AD amongst pregnant women. The experience of a previous obstetric complication is known to increase the likelihood of recurrence, a fact that is emphasised to women at the time of debriefing them to encourage them to seek care early to mitigate the recurrence and their consequences. This becomes an additional psychological stressor to the already vulnerable woman on account of the pregnancy itself, thus predisposing to depression early in pregnancy.^58^ As the present study was limited to early pregnancy, and most obstetric complications occur in latter pregnancy by definition, we were unable to provide valid analysis on the effect of current obstetric complications variables such as bleeding, pain, cervical incompetence and medical disorders such as anaemia, hypertension, diabetes and hypothyroidism.

The major strength of this study is the use of geographically and religiously diverse population as a representation of national demographics, which can potentially improve the generalisation of the results. The sample size is also higher than those of previous studies for the country. However, the result of this study should be interpreted with regard to certain limitations. Being a cross-sectional study, causality cannot be established, and further longitudinal studies are required to understand the nature of the risk factors for AD identified in this study. Moreover, this study is facility-based; hence, pregnant women not seeking antenatal services were not represented. Further, the use of EPDS screening tool could account for differences in prevalence figures between our study and reports from settings where the study design differs by this screening tool. Care should also be taken in generalising the results to antenatal women in latter half of pregnancy, as this study only included women in early half of pregnancy. In addition, as this study was conducted in private facilities, the findings might not be generalisable to women attending public institutions. Finally, antenatal depressive symptoms were assessed using self-reported questionnaires, which was a screening rather than a diagnostic tool, and with the possibility for recall bias.

## Conclusion

Nearly one out of three pregnant women in their first half of pregnancy attending antenatal services at private health facilities in Southern Eastern and South Western Nigeria showed depressive symptoms on screening. Low socio-economic status, previous history of pregnancy complication, unplanned current pregnancy, past history of depression, IPV, marital dissatisfaction, poor social support and foetal male-gender preference were found to be significant risk factors for the AD. There is the need to urgently address mental health issues and incorporate psychiatric services into antenatal care programmes. The aforementioned risk factors should be incorporated into screening and intervention strategy to effectively reduce the burden of AD. Moreover, existing government and other stakeholder interventions aimed at addressing the risk factors needs to be evaluated in the light of present findings to help reduce the burden of AD. Further studies are needed to explore individual risk factors to help make recommendations on the most optimal way of addressing each of the factors.
